# How Anxiety Shapes Students’ Self-Rated Health at Elite Universities: A Longitudinal Study

**DOI:** 10.3390/bs16020197

**Published:** 2026-01-29

**Authors:** Xinqiao Liu, Xinyuan Zhang, Yuyang Liu

**Affiliations:** 1 School of Education, Tianjin University, Tianjin 300350, China; 2 Department of Alumni and Foundation Affairs, University of International Business and Economics, Beijing 100029, China

**Keywords:** anxiety, self-rated health, cross-lagged model, mental health, elite colleges

## Abstract

Self-rated health is a comprehensive indicator reflecting an individual’s subjective assessment of their overall health status. The health condition of students in elite universities is directly related to the quality of talent reserves and the long-term development of the country. However, the multiple challenges they face make them prone to subhealth issues. To understand and effectively intervene in the health dilemmas of this group from a psychological perspective, this study constructed a cross-lagged model to examine the potential bidirectional relationship between anxiety and self-rated health. We utilized two-wave longitudinal data from a sample of 896 undergraduate students (mean age 21.37 years, 60.27% male, 92.08% Han nationality) from five elite universities in Beijing, China. Anxiety was measured using the Depression Anxiety Stress Scales, while self-rated health was assessed via a single-item score. The study revealed that during the two survey periods, the anxiety levels of elite university students decreased (7.682/7.462), whereas their self-rated health scores increased (81.781/83.255). Higher levels of anxiety were significantly associated with lower levels of self-rated health in both the concurrent and cross-lagged analyses (r = −0.299~−0.173, *p* < 0.01). Prior anxiety could predict later self-rated health (*β* = −0.081, *p* < 0.05), but the reverse path from self-rated health to anxiety was not confirmed. Our findings indicate that anxiety among elite university students has a unidirectional prospective effect on self-rated health. On the basis of these findings, universities should integrate mental health services into their routine work systems, and students should also increase their sense of personal responsibility for their own health, actively seeking effective pathways to improve their physical and mental well-being.

## 1. Introduction

Enhancing the health level of the population is an inevitable requirement for achieving global public health goals. In this context, the physical and mental health of college students, as the backbone of the nation’s future development, is of paramount importance. However, compared with the general population, college students are more susceptible to health issues, particularly mental health problems (Hsu & Chiang, 2020; J. Zhang et al., 2024; Cao & Liu, 2025, 2022). Previous research has shown that students in nonhealth-related departments have relatively lower levels of health literacy (Rababah et al., 2019; S. C. Yang et al., 2017). Moreover, college students now possess greater decision-making autonomy than before, leading to greater risks of engaging in unhealthy behaviors (e.g., smartphone addiction, lack of exercise, alcohol and drug use) (Mei et al., 2023), which may mildly affect academic performance (Ruthig et al., 2011) or severely increase future disease risk, even endangering lives (Arsandaux et al., 2020; X. Q. Liu & Wang, 2024b). For students in elite institutions, this issue is even more pronounced, and the potential consequences are more severe. They hold high expectations from families, schools, and society and are entrusted with hopes in terms of academic achievements, interpersonal relationships, career development, and even personal value realization (C. J. Yang et al., 2021). This unique, chronic stress may subject them to unprecedented burdens, making them a particularly vulnerable group prone to anxiety and health risks (Kabiri et al., 2024; Cooper et al., 2018). Therefore, it is crucial to study the psychological and health issues of students at elite universities.

Notably, college students are actively seeking more information about maintaining or improving their health (D. G. Zhang et al., 2021; Bak et al., 2022; Patil et al., 2021). Self-rated health (SRH) is a comprehensive health measurement indicator that effectively reflects an individual’s subjective assessment of their physical and mental health and functioning (Chow et al., 2018). Essentially, SRH is an individual subjective cognitive process embedded within specific sociocultural contexts. Since health itself lacks a unified direct measurement standard, individuals must autonomously select information they deem relevant to health as the basis for assessment (Jylhä, 2009). Traditional views suggest that SRH is significantly associated with a range of factors, such as self-esteem (X. Q. Liu et al., 2024a), health literacy (Nie et al., 2021), employment status (Ge et al., 2021), social relationships (H. L. Yang et al., 2021), and physical activity (Pierannunzio et al., 2022). Additionally, the negative impacts of public health emergencies (Tusl et al., 2021), exposure to adverse environments (Hautekiet et al., 2022; Z. M. Yang et al., 2022), and other factors are also linked to lower SRH levels. Among the numerous associated factors, psychological factors, particularly anxiety, may play a significant yet underappreciated role. Anxiety is one of the most prevalent and severe psychological issues among college students in China and globally and is characterized by diverse developmental trajectories and impact mechanisms (Hoyt et al., 2021; Mohammed et al., 2021; Li et al., 2022; X. Q. Liu & Zhang, 2026). Compared to other mental factors, anxiety—as a fundamental negative emotional state—is more readily identifiable and measurable, serving as an entry point for early psychological risk warning and intervention. From a psychological and behavioral perspective, anxiety not only directly induces negative emotional experiences but also triggers a series of health-damaging behaviors (Stanton et al., 2020). These behavioral changes impair physiological health and psychological functioning, thereby affecting individuals’ overall assessment of their health status. Therefore, clarifying anxiety’s impact on SRH represents a crucial and feasible entry point for understanding and intervening in college students’ health challenges.

### 1.1. Literature Review

Mental health and SRH are often included together as outcome variables to examine the impact of a certain factor on health (Marchiondo et al., 2019; Oftedal et al., 2019; Bekalu et al., 2019; Porru et al., 2022). Previous empirical studies have also shown a significant association between mental health and SRH, with the form of association and mechanisms of action exhibiting diverse characteristics. Windsor et al. (2016) reported that SRH was negatively correlated with mental health and that social network characteristics could buffer the negative impact of declining self-rated health on mental health to some extent. Similarly, in the UK Million Women Study, Liu et al. reported a negative association between poor SRH and well-being and further indicated that SRH was an important adjustment factor in explaining the relationship between unhappiness and mortality (B. Liu et al., 2016). When the contributions of physiological, psychological, and socioeconomic factors to SRH are assessed, mental health has been proven to be the most significant predictor of SRH (Hamplová et al., 2022). Craig et al. (2018) supplemented the mediation mechanism, finding that mental health not only has a direct positive association with SRH but also plays a mediating role in the relationship between health behaviors and SRH. A multinational cross-sectional study similarly supported this conclusion, showing that older adults with poorer mental health were more likely to report lower SRH, a result that was consistent across both China and the United States (Xu et al., 2019). However, some studies have reported racial differences in the correlation between mental health issues and SRH (Assari, 2017; Jang et al., 2014). Beyond unidirectional effects, some research has begun to explore the bidirectional mechanisms between mental health and SRH further. For example, a study investigating the relationships between socioeconomic status, SRH, and mental health demonstrated that mental health and SRH mutually predict each other and mediate the relationship between socioeconomic status and the other, confirming the existence of a bidirectional influence mechanism (Achdut & Sarid, 2020).

Anxiety is an emotional state arising from uncertainty about potential future threats, representing one of the most common mental health issues (Grupe & Nitschke, 2013). Numerous studies have revealed significant prevalence rates of anxiety among college students. The Spring 2022 National College Health Assessment in the United States indicated that 34.60% of students had been previously diagnosed with an anxiety disorder (Tan et al., 2023). In a sample of Spanish college students, 23.6% reported experiencing anxiety symptoms (Ramón-Arbués et al., 2020). More importantly, anxiety among college students exhibits distinct fluctuation patterns across academic stages (X. Q. Liu et al., 2023). In the absence of effective interventions, it may trigger a series of negative consequences, including poor academic performance (BlackDeer et al., 2023) and sleep disorders (Boehm et al., 2016).

According to previous research, anxiety may have a complex bidirectional relationship with SRH. On the one hand, anxiety is a significant predictor of SRH, with individuals who experience higher levels of anxiety typically reporting lower SRH scores (Fink et al., 2010; Washington et al., 2018). For example, using a multilevel model, Kim and Katelyn Kim (2021) reported that emotional anxiety triggered by the COVID-19 pandemic had a negative predictive effect on SRH. On the other hand, SRH can also predict anxiety. One study indicated that anxiety is a predictor of poor SRH among women, with a significant positive correlation (Rouch et al., 2014). Another study verified the ability of SRH to predict anxiety levels three years later, as well as in cases where anxiety symptoms reached critical thresholds (Östberg & Nordin, 2022). Research has also revealed interaction patterns between two variables through mediating mechanisms. The current literature proposed anxiety as a mediator between variables such as sleep quality (Zhu et al., 2023), cognitive behavioral therapy (Hedman-Lagerlöf et al., 2017), perceived discrimination (Carter et al., 2016), and health status, with the alleviation of anxiety being key to improving SRH. Conversely, SRH has a significant negative impact on negative emotions, including anxiety, and plays a mediating role in the process through which physical exercise promotes mental health (Mu et al., 2024). Similarly, the relationship between changes in intimate trust and trait anxiety is also mediated by SRH (Jhang, 2020). Additionally, SRH can indirectly affect health-related quality of life (Fang et al., 2024) and physical activity (Huang et al., 2025) through single or sequential mediation by anxiety. More innovatively, SRH and anxiety symptoms can jointly form a serial mediation pathway to explain more complex mechanistic issues. A typical example is studies explaining the mechanism of the impact of hearing loss on cognitive function, where regardless of the direction of SRH and anxiety, they constitute a sequential mediation pathway, clearly elucidating this influence process (Chen et al., 2023).

### 1.2. Theoretical Framework

For a long time, emotion, behavior, and cognition have been regarded as the core internal processes of human psychology. Functionalism posits that mental states and behaviors are formed to adapt to environmental demands, a perspective that helps unify the three as mechanisms serving specific goals in particular environments (Lench et al., 2013). Applying this framework, this study aims to explore how students’ anxiety experiences and health perceptions influence each other within the specific high-pressure, highly competitive environment of elite universities, collectively constituting their psychological adaptation process in coping with environmental challenges. From the cognitive-behavioral theory of anxiety, anxiety may trigger attentional and interpretive biases toward potential threats (Cisler & Koster, 2010) and promote behavioral avoidance (Visu-Petra et al., 2013), thereby impairing health perceptions. On the other hand, concerns about health status can themselves become a stressor, triggering or exacerbating anxiety. Therefore, there is an urgent need to investigate the relationship between anxiety and SRH.

### 1.3. The Present Study

Research on the relationship between anxiety and SRH has evolved into a systematic exploration encompassing unidirectional predictions and mediating pathways, yet unresolved issues remain. First, the bidirectional predictive relationship between anxiety and SRH has been confirmed by existing studies, but most studies employ cross-sectional data from a single time point. Although some studies have attempted to track the long-term predictive power of SRH for anxiety, the temporal mechanisms of their bidirectional relationship still require further validation through more longitudinal research. Second, previous studies have focused particularly on two groups—healthcare-related personnel and the elderly—with a notable lack of research targeting university students, especially those from elite institutions. Given that the macrolevel background of a nation’s development is critical, students from elite universities hold the important mission of precultivation, and their health status is directly related to the quality of talent and the sustainability of national development. In this sense, clarifying the relationship between anxiety and SRH is crucial for understanding the health challenges faced by this group and addressing their health needs.

**Figure 1:**
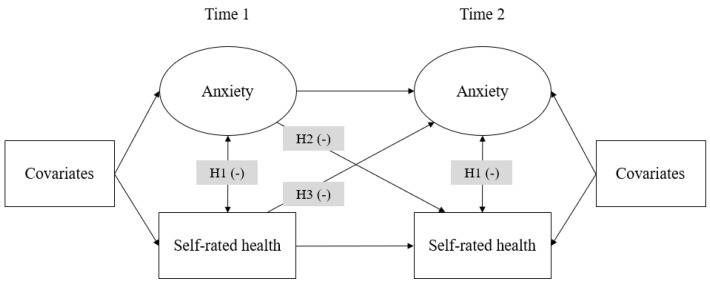
Research theoretical framework. The negative sign in parentheses denotes a negative correlation or negative predictive relationship.

On the basis of the aforementioned literature review, this study aims to investigate the relationship between anxiety and SRH through a longitudinal design among students in China’s elite universities (Figure 1). Specifically, we utilize two-wave survey data to examine the bidirectional predictive effects of anxiety and SRH across two time points. Drawing from prior research, we propose the following three hypotheses:

**Hypothesis** **1.** *There is a significant negative correlation between anxiety and SRH among Chinese elite university students.*

**Hypothesis** **2.** *Anxiety has a significant negative predictive effect on SRH among Chinese elite university students.*

**Hypothesis** **3.** *SRH has a significant negative predictive effect on anxiety among Chinese elite university students.*

## 2. Materials and Methods

### 2.1. Participants and Procedure

Our data come from a longitudinal growth tracking survey conducted among college students in Beijing, China. The study subjects were undergraduate students from five Project 985 universities in Beijing, including Tsinghua University, Peking University, Renmin University of China, Beihang University, and the Beijing Institute of Technology. Project 985 universities represent the highest level of higher education in China, occupy the premier tier of the higher education system, and are widely recognized as elite higher education institutions (L.-f. Zhang et al., 2022). Participants were selected using probability proportional to size sampling. This study involved two rounds of questionnaire surveys conducted at Time 1 (T1) and Time 2 (T2) with one-year intervals. Specifically, the baseline survey was conducted in June 2009, with participants in their third year of undergraduate studies. The follow-up survey was completed between May and July 2010, at the end of their fourth undergraduate year. A total of 896 students completed the baseline survey at T1. At T2, a follow-up survey was conducted with the same group of participants, and 809 valid samples were successfully collected, with an attrition rate of 9.71%. Further *t* tests comparing lost samples and retained samples on key variables such as gender and age yielded results consistent with random attrition characteristics.

### 2.2. Ethical Consideration

All participants read and signed informed consent forms prior to the survey commencement. The research process strictly adhered to the following ethical principles: a. Confidentiality: All collected data was used exclusively for this study and was securely stored by the researchers. b. Voluntariness: Participants were explicitly informed that their involvement was entirely voluntary. c. Anonymity: The questionnaires were administered anonymously, and no personally identifiable information was used during data analysis or results reporting. d. Right to Withdraw: Participants could withdraw from the survey at any stage without incurring any adverse consequences.

### 2.3. Power Consideration

This study employed GPower 3.1.9.7 to conduct a priori sample size calculation and post hoc power analysis. Based on previous research, it was hypothesized that the correlation coefficient between the two core variables in this study would range from −0.42 to −0.19. The priori results indicated that the minimum required sample size was 349 participants with the 5% significant level (two-tailed) and a power (1 − β) of 95%. Post hoc power analysis revealed that our sample size (N = 896) could provide a power of 99.99% for estimating a correlation coefficient of −0.19 at the 5% significance level.

### 2.4. Measures

#### 2.4.1. Anxiety

Anxiety was measured via the Depression Anxiety Stress Scale (DASS) subscale. The DASS is a classic psychometric tool, and its reliability and cross-cultural validity have been confirmed (Crawford & Henry, 2003; Moussa et al., 2017). This study employed the Chinese translated version of the DASS-Anxiety, which has been used and validated by multiple Chinese scholars (X. Q. Liu & Wang, 2024a). The subscale consists of 14 items, requiring participants to assess the degree to which each description matches their own situation on the basis of the previous week (e.g., “I felt my mouth was dry”). The scale uses a 4-point Likert scoring method, with scores ranging from 0 (“Not at all applicable”) to 3 (“Very applicable, or most applicable”). Higher total scores indicate more severe anxiety levels. The Cronbach’s alpha values in the first and second rounds of the survey were 0.863 and 0.877, respectively, indicating good reliability of the anxiety scale.

#### 2.4.2. Self-Rated Health

In this study, SRH was measured through a self-reported single-item questionnaire. Specifically, participants were asked to rate their current overall health status on a scale from 0 to 100, with higher scores indicating better perceived health. The use of a single-item measure for SRH effectively reflects an individual’s holistic perception of health and is one of the most commonly used indicators in health research (Fayers & Sprangers, 2002). This item was phrased in Chinese, and its validity has been supported by studies conducted among Chinese populations (Cai et al., 2017).

#### 2.4.3. Other Covariates

In this study, sociodemographic information, including gender (male, female), age, ethnicity (Han nationality, national minority), personality, and family social status (upper, upper-middle, middle, lower-middle, lower), was introduced as a covariate in the model to control for potential confounding factors and test the robustness of the results. Covariates were all based on self-reported data from T1.

### 2.5. Data Analysis

Data processing and model testing in this study were performed via Stata 15.0 and Mplus 7.4. First, we used Stata to conduct descriptive and correlational analyses on anxiety and SRH at T1 and T2, laying the foundation for subsequent in-depth analyses. Next, we constructed a cross-lagged model to examine the bidirectional relationship between anxiety and SRH. The cross-lagged model serves to examine the forward-looking predictive effect of one variable on another while controlling for correlations between variables at the same time point and the cross-temporal stability of the variables themselves (Orth et al., 2021).

**Figure 2:**
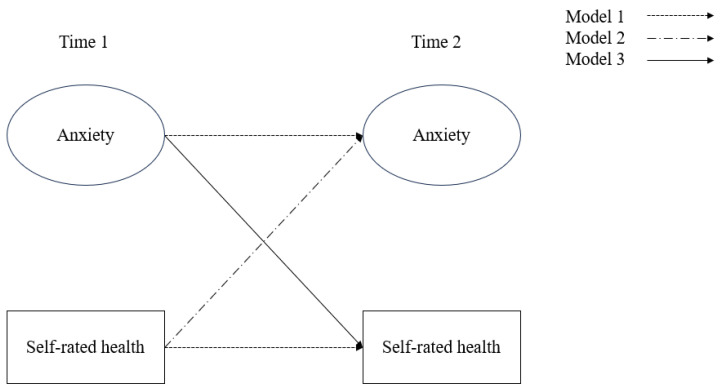
Cross-lagged models between anxiety and self-rated health.

The models constructed in this study are shown in Figure 2. Model 1 (M1) is an autoregressive model containing only anxiety and SRH, without cross-lagged paths. The role of this model is to test the stability of the two variables. Model 2 (M2) adds a cross-lagged path from SRH at T1 to anxiety at T2 on the basis of the autoregressive paths, with a focus on testing the predictive effect of prior SRH on later anxiety. Model 3 (M3) adds a cross-lagged path from anxiety at T1 to SRH at T2 on the basis of the autoregressive paths, with a focus on testing whether prior anxiety affects later SRH. Model 4 (M4) includes all paths from Models 1–3 to examine the bidirectional relationship between anxiety and SRH. Model 5 (M5) incorporates covariates such as gender, age, ethnicity, personality, and family social status on the basis of Model 4 to investigate the effects of covariates at T1 on anxiety and SRH at T1 and T2. Finally, the optimal model is selected through comparison.

The model fit evaluation in this study was conducted primarily through five indicators: the chi-square value, the root mean square error of approximation (RMSEA), the standardized root mean square residual (SRMR), the comparative fit index (CFI), and the Tucker–Lewis index (TLI). According to Hu and Bentler (1999), a model demonstrates an excellent fit when RMSEA ≤ 0.06, SRMR ≤ 0.08, CFI ≥ 0.95, and TLI ≥ 0.95. Additionally, a model is considered acceptable when RMSEA < 0.10, SRMR < 0.10, CFI > 0.9, and TLI > 0.9.

## 3. Results

### 3.1. Descriptive Statistics and Correlation Analysis

The sociodemographic characteristics of the sample students are shown in Table 1. 60.27% were male, 92.08% were of Han nationality, 32.48% had siblings, and 75.33% resided in urban areas. The average age of the participants at T1 was 21.37 years, with a standard deviation of 0.89. The average personality tendency score was 5.50, with a standard deviation of 1.65 (numbers 1 to 9 represent the degree from introversion to extroversion).

Table 2 presents the descriptive statistics and correlation analysis results of the two core variables. A total of 896 participants were enrolled at T1, among whom 809 were retained at T2. The mean anxiety scores measured at T1 and T2 were 7.682 (SD = 6.058) and 7.462 (SD = 6.174), respectively, while the mean SRH scores were 81.781 (SD = 8.784) and 83.255 (SD = 8.377), respectively. This indicates a slight decrease in overall anxiety levels and an increase in SRH within the sample. According to the DASS-42 user manual, anxiety scores ranging from 0 to 7 are considered normal, while scores between 8 and 9 indicate mild (Lovibond & Lovibond, 1995). In this study, anxiety scores were consistently near the upper limit of the normal range, approaching the threshold for mild anxiety. The results of the correlation analysis revealed significant negative correlations between anxiety and SRH both concurrently and across time periods. Specifically, anxiety at T1 was negatively correlated with SRH at T1 (r = −0.231, *p* < 0.01), anxiety at T2 was negatively correlated with SRH at T2 (r = −0.299, *p* < 0.01), anxiety at T1 was negatively correlated with SRH at T2 (r = −0.205, *p* < 0.01), and anxiety at T2 was negatively correlated with SRH at T1 (r = −0.173, *p* < 0.01). A stable and significant negative association was observed between anxiety and SRH among elite university students, confirming Hypothesis 1.

### 3.2. Model Parameters

We validated the five models separately, and their fitting conditions are shown in Table 3, with the path coefficients presented in Table 4. Overall, all the models met the acceptable fitting standards: the RMSEA values ranged between 0.06 and 0.07, the SRMR values ranged between 0.01 and 0.04, and both the CFI and TLI were greater than 0.9 (mostly exceeding 0.95).

Specifically, M1 demonstrated a good fit in the autoregressive paths of anxiety and SRH (RMSEA = 0.075, SRMR = 0.033, CFI = 0.972, TLI = 0.957). The autoregressive effects of anxiety (*β* = 0.686, *p* < 0.05) and SRH (*β* = 0.583, *p* < 0.05) were both significant, indicating high stability of these two variables. M2 showed a good fit (RMSEA = 0.077, SRMR = 0.029, CFI = 0.972, TLI = 0.954), but compared with M1, the difference in X^2^ was not significant (∆X^2^ = 1.427, *p* > 0.05). Additionally, the cross-lagged path from SRH at T1 to anxiety at T2 was not significant (*β* = −0.038, *p* > 0.05), suggesting that the lagged effect of SRH on anxiety was not confirmed. M3 exhibited a good fit (RMSEA = 0.074, SRMR = 0.021, CFI = 0.974, TLI = 0.957), and the comparison with M1 was significant (∆X^2^ = 7.424, *p* < 0.05). The cross-lagged path from anxiety at T1 to SRH at T2 was statistically significant (*β* = −0.093, *p* < 0.05), demonstrating that anxiety negatively predicts SRH.

M4 is an integrated model that includes an autoregressive path and two cross-lagged paths, demonstrating good fit indices (RMSEA = 0.076, SRMR = 0.019, CFI = 0.974, TLI = 0.955). The comparison with M1 yielded significant results (∆X^2^ = 8.385, *p* < 0.05). Similarly to the findings from the previous three models, anxiety and SRH were stable across different time periods in M4. The lagged effect of SRH at T1 on anxiety at T2 was not significant (*β* = −0.031, *p* > 0.05), whereas the lagged effect of anxiety at T1 on SRH at T2 was significant (*β* = −0.090, *p* < 0.05). These results further confirm that prior anxiety levels significantly and negatively influence subsequent SRH status, but the reverse predictive relationship was not statistically supported. This model achieves a relatively better balance between model fit and parsimony, making it the optimal model for explaining the relationship between anxiety and SRH.

**Figure 3:**
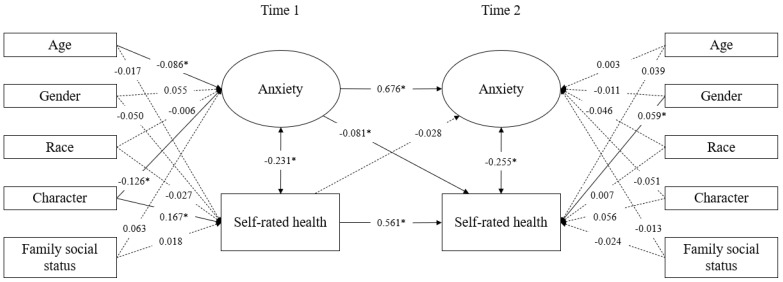
Model (M5) of anxiety and self-rated health with standardized coefficients. * 5% significance level.

To test the robustness of the model, we further designed M5, which incorporates multiple covariates. Although some fit indices slightly decreased, they still showed acceptable fit (RMSEA = 0.063, SRMR = 0.020, CFI = 0.962, TLI = 0.929). The path coefficients and significance of this model were also similar to those of the previous models. Therefore, the main relationship pattern between anxiety and SRH among Chinese elite university students remained stable after controlling for potential confounding factors (Figure 3). These results confirm Hypothesis 2 but do not confirm Hypothesis 3.

## 4. Discussion

In current society, health issues among university students cannot be overlooked (Auerbach et al., 2018; Son et al., 2020; X. Liu et al., 2023). Owing to the uniqueness and importance of students in elite universities, their physical and mental health conditions have a far-reaching impact. On the basis of the findings of this study, we can delve deeper into the relationship between anxiety and SRH within this group, providing empirical support for effectively addressing health concerns.

Descriptive statistical data revealed that, compared with those at T1, the anxiety levels of elite university students were lower at T2, whereas their SRH scores were higher. Notably, the baseline survey of this study was conducted during the junior year. In other words, from the junior year to the senior year, students’ anxiety levels slightly decreased, and their SRH scores slightly increased. This may be attributed to the clarification of academic and career prospects, lifestyle changes, and improvements in psychological adaptation and adjustment capabilities. Specifically, upon entering the senior year, most students have clarified their development directions (further education or employment) and have more discretionary time to optimize their lifestyles. Moreover, students’ psychological adjustment abilities, such as emotional regulation and stress coping, gradually strengthen, positively influencing anxiety alleviation and health improvement (X. Q. Liu et al., 2024b). The results of the correlation analysis revealed a significant negative relationship between anxiety and SRH, further supporting previous research findings (Chen et al., 2023; Hedman-Lagerlöf et al., 2017; Huang et al., 2025). In other words, as individuals’ anxiety levels increase, their SRH scores tend to decrease.

In the cross-lagged model, we found a unidirectional influence mechanism between anxiety and SRH, indicating that anxiety has a prospective effect on SRH. Specifically, lower levels of anxiety predict higher SRH scores one year later, whereas higher levels of anxiety predict lower SRH scores one year later. This unidirectional relationship provides a new theoretical perspective for understanding the formation mechanisms of subjective health perceptions in specific high-pressure environments. From an integrated framework of functionalism and cognitive-behavioral theory, this anxiety-to-health perception pathway may represent an adaptive cost incurred by elite university students when coping with environmental stressors. Studies have shown that individuals with anxiety exhibit attentional biases, where their attention is more likely to be directed toward threatening and negative information (Cisler & Koster, 2010; Van Bockstaele et al., 2014). This cognitive pattern may lead them to excessively focus on minor bodily changes or temporary symptoms and interpret them as signs of serious health issues, thereby resulting in negative self-assessments of their health status. Additionally, some studies have reported that anxiety is accompanied by declines in executive function and behavioral avoidance (Visu-Petra et al., 2013). Individuals in an anxious state often struggle with maintaining goals and flexible coping strategies and are more likely to adopt avoidance behaviors in health-promoting activities such as physical exercise and social interactions. In the long term, this can harm their physical health and reinforce their negative evaluations of health.

However, the relatively small predictive effect size observed in this study holds significant theoretical value. It may suggest that among high-achieving groups such as elite university students, the negative pathway from anxiety to SRH might be buffered by certain factors. One hypothesis we propose is that the strong cognitive abilities, academic self-efficacy, and problem-solving tendencies typically possessed by this group constitute important psychosocial resources. These resources partially counteract the negative spillover effects of anxiety. Therefore, the small effect size captured in this study may reflect a boundary condition of the stress–health model in high-resource populations. In other words, protective factors moderate the ultimate impact intensity of risk factors. This theory awaits verification in future research.

In this study, we also found that the predictive effect of prior SRH on anxiety one year later was not significant, which seems to contradict the findings reported in previous studies (Östberg & Nordin, 2022). This discrepancy may stem from multiple underlying reasons. First, age is a critical factor influencing SRH assessments (Jylhä, 2009). Compared with elderly individuals, college students are generally in a phase of rising or peaking physiological function, with a far lower risk of chronic or severe illnesses. Their SRH evaluations primarily reflect short-term, temporary health issues (e.g., colds, fevers, and sleep disturbances) rather than permanent functional decline. This represents a fundamental departure from existing research, which has focused primarily on the elderly population. Second, the primary sources of anxiety among elite university students are concerns about academic performance, achievement goals, career development, and financial pressure—issues directly tied to college life and personal growth (Beiter et al., 2015). Clearly, short-term health problems do not directly trigger long-term anxiety among students. Finally, from a methodological perspective, the single-item SRH measurement used in this study focuses more on overall health judgments and may inadequately capture specific symptoms closely linked to anxiety. Additionally, this approach cannot control which aspects health respondents emphasize during self-assessment (Jylhä, 2009), potentially leading to an underestimation of the relationship between anxiety and SRH.

Health is an eternal proposition that runs through life. This study explores the related factors and predictive pathways of health status from the perspective of mental health, aiming to provide theoretical references and insights for addressing health issues among elite university students and improving their health conditions. As the main front of education, universities must attach great importance to students’ mental health issues. They should fully leverage their platform role in psychological intervention and services and establish systematic and professional support systems. On the one hand, universities need to integrate multiple resources to construct a multilevel, interconnected mental health service network. On the other hand, digital technologies and artificial intelligence tools should be actively incorporated into mental health services. It is also essential to establish early screening mechanisms for psychological issues and provide targeted interventions such as cognitive behavioral therapy and mindfulness-based therapies for high-risk students. From the student perspective, it is essential to strengthen awareness of taking primary responsibility for their own health and improve their self-regulation capabilities to reduce the generation and exacerbation of anxiety. By engaging in physical exercise, actively participating in social interactions, and cultivating good habits, students can optimize their lifestyles and promote the healthy development of both the body and their minds. In addition, they should abandon stigmatized perceptions of mental health issues, proactively seek relevant services, and build internal defenses to safeguard their own health.

## 5. Limitations and Future Directions

This study has certain limitations in terms of its design and measurement. First, all the core variables rely on self-reports from the research subjects. Although questionnaires are commonly used in social science research, there may be issues of measurement bias. Second, despite the widespread use of single-item SRH measurement in sociological health research and public health studies, this approach faces limitations in controlling the specific health dimensions and subjective weightings that respondents rely on when self-rating. Consequently, it may compromise the content validity of SRH indicators. Third, the cross-lagged panel model in this study involves only two time points, limiting the in-depth exploration of more complex and dynamic interactions between anxiety and SRH. Fourth, the model did not incorporate potential confounding variables such as academic stress, social support, or frequency of physical exercise. These factors may simultaneously influence both anxiety and SRH, thereby potentially affecting the interpretation of the results. Fifth, the sample in this study exhibits uneven distribution across ethnic groups and urban-rural origins. While this sample structure reflects certain typical characteristics of student populations at China’s elite universities to some extent, it may also limit the generalizability of the findings.

Overall, future research could deepen and expand in the following aspects. With respect to research design, a mixed-methods approach should be employed, and additional waves of data collection should be incorporated to capture more profound changes. With respect to measurement methods, multidimensional measurement tools should be employed, such as subjective SRH with objective health indicators. Concurrently, tests of temporal measurement stability should be conducted on scales to enhance the reliability, validity, and comparability. In terms of mechanism exploration, potential mediating and moderating pathways should be further tested, and the conclusions of this study should be validated in broader non-elite university populations and multicultural contexts.

## 6. Conclusions

This study systematically examined and validated the longitudinal relationship between anxiety and self-rated health via a longitudinal survey of students at elite universities in China. First, there is a significant and stable negative correlation between anxiety and SRH. Second, anxiety can longitudinally predict SRH, but the reverse effect of SRH on anxiety has not been substantiated. These findings have multiple implications. At the theoretical level, they deepen our understanding of the complex dynamic relationship between mental health and subjective health evaluations, particularly providing a theoretical framework for research on important groups of elite university students. Practically, the conclusions provide clear directions for universities to optimize mental health service systems and promote proactive health management among students.

## Figures and Tables

**Table 1:** Characteristics of the participants.

Variable	Category	Number	Percent	M ± SD	Range
Gender	Female	356	39.73%		
	Male	540	60.27%		
Age		896		21.37 ± 0.89	18–27
Nationality	Han nationality	825	92.08%		
	National minority	71	7.92%		
Character		896		5.50 ± 1.65	1–9
Sibling	Yes	291	32.48%		
	No	605	67.52%		
Hometown location	Urban	675	75.33%		
	Rural	221	24.67%		
Father’s education level	Never accept formal education	4	0.49%	13.61 ± 3.60	0–19
	Primary school	36	4.45%		
	Middle school	135	16.69%		
	Senior high school	198	24.47%		
	Bachelor’s degree or associate degree	360	44.50%		
	Master’s degree and above	76	9.39%		
Mother’s education level	Never accept formal education	23	2.86%	12.49 ± 4.02	0–19
	Primary school	70	8.71%		
	Middle school	121	15.05%		
	Senior high school	244	30.35%		
	Bachelor’s degree or associate degree	324	40.30%		
	Master’s degree and above	22	2.74%		
Family economic status	Upper	4	0.45%		
	Upper-middle	159	17.75%		
	Middle	431	48.10%		
	Middle-low	244	27.23%		
	Low	58	6.47%		
Family social status	Upper	9	1.00%		
	Upper-middle	238	26.56%		
	Middle	418	46.65%		
	Middle-low	172	19.20%		
	low	59	6.58%		

Note: The educational attainment of the father and mother is measured by the number of years of education. The mean represents the average number of years of education for the parents, and the range refers to the fluctuation interval of the years of education. The specific assignment methods are as follows: no formal education is 0, elementary school is 6, junior high school is 9, high school (including vocational high school and technical secondary school) is 12, university (including college) is 16, and postgraduate or above is 19.

**Table 2:** Descriptive statistics and correlations for the main variables.

Variables	Mean	Standard Deviation	1	2	3	4
1. Anxiety-T1	7.682	6.058	1			
2. Self-Rated Health-T1	81.781	8.784	−0.231 ***	1		
3. Anxiety-T2	7.462	6.174	0.582 ***	−0.173 ***	1	
4. Self-Rated Health-T2	83.255	8.377	−0.205 ***	0.589 ***	−0.299 ***	1

Note: The correlation matrix reports Pearson correlation coefficients. *** *p* < 0.01.

**Table 3:** Fit indices of the models.

Model	X^2^	df	RMSEA [90% CI]	SRMR	CFI	TLI	Comparison	∆X^2^	*p*
Model 1	109.055	18	0.075 [0.062–0.089]	0.033	0.972	0.957			<0.05
Model 2	107.628	17	0.077 [0.063–0.091]	0.029	0.972	0.954	M1-M2	1.427	>0.05
Model 3	101.631	17	0.074 [0.061–0.088]	0.021	0.974	0.957	M1-M3	7.424	<0.05
Model 4	100.670	16	0.076 [0.063–0.091]	0.019	0.974	0.955	M1-M4	8.385	<0.05
Model 5	159.317	36	0.063 [0.053–0.073]	0.020	0.962	0.929	M1-M5	−50.262	<0.05

**Table 4:** Overview of the standardized stability and cross-lagged coefficients.

Model	Autoregressive Path	*β*	Cross-Lagged Path	*β*
Model 1	Anxiety (T1)→Anxiety (T2)	0.686 *		
	Self-Rated Health (T1)→Self-Rated Health (T2)	0.583 *		
Model 2	Anxiety (T1)→Anxiety (T2)	0.675 *	Self-Rated Health (T1)→Anxiety (T2)	−0.038
	Self-Rated Health (T1)→Self-Rated Health (T2)	0.588 *		
Model 3	Anxiety (T1)→Anxiety (T2)	0.698 *	Anxiety (T1)→Self-Rated Health (T2)	−0.093 *
	Self-Rated Health (T1)→Self-Rated Health (T2)	0.561 *		
Model 4	Anxiety (T1)→Anxiety (T2)	0.689 *	Self-Rated Health (T1)→Anxiety (T2)	−0.031
	Self-Rated Health (T1)→Self-Rated Health (T2)	0.566 *	Anxiety (T1)→Self-Rated Health (T2)	−0.090 *
Model 5	Anxiety (T1)→Anxiety (T2)	0.676 *	Self-Rated Health (T1)→Anxiety (T2)	−0.028
	Self-Rated Health (T1)→Self-Rated Health (T2)	0.561 *	Anxiety (T1)→Self-Rated Health (T2)	−0.081 *

Note: * 5% significance level.

## Data Availability

The original contributions presented in this study are included in the article. Further inquiries can be directed to the corresponding authors.

## Abbreviations

The following abbreviations are used in this manuscript:

SRH	Self-rated health
DASS	Depression Anxiety Stress Scale
